# Cognitive Neural Mechanism of Backward Inhibition and Deinhibition: A Review

**DOI:** 10.3389/fnbeh.2022.846369

**Published:** 2022-05-20

**Authors:** Jiwen Chen, Shujie Wu, Fuhong Li

**Affiliations:** School of Psychology, Jiangxi Normal University, Nanchang, China

**Keywords:** backward inhibition, deinhibition, task switching, n-2 repetitive costs, cognitive control

## Abstract

Task switching is one of the typical paradigms to study cognitive control. When switching back to a recently inhibited task (e.g., “A” in an ABA sequence), the performance is often worse compared to a task without N-2 task repetitions (e.g., CBA). This difference is called the backward inhibitory effect (BI effect), which reflects the process of overcoming residual inhibition from a recently performed task (i.e., deinhibition). The neural mechanism of backward inhibition and deinhibition has received a lot of attention in the past decade. Multiple brain regions, including the frontal lobe, parietal, basal ganglia, and cerebellum, are activated during deinhibition. The event-related potentials (ERP) studies have shown that deinhibition process is reflected in the P1/N1 and P3 components, which might be related to early attention control, context updating, and response selection, respectively. Future research can use a variety of new paradigms to separate the neural mechanisms of BI and deinhibition.

## Introduction

Backward inhibition (BI) refers to the continued inhibition from a recently performed task (Grange and Houghton, 2010; Costa and Friedrich, 2012). Mayr and Keele (2000) first adopted the sequence task switching paradigm and revealed the BI effect, also termed as the N-2 repetition costs (Mayr and Keele, 2000; Lien and Ruthruff, 2008; Philipp et al., 2008; Koch et al., 2010). BI in task switching can be explained as follows: when switching to a task that has just been executed (e.g., ABA task sequence), it takes longer response time than switching to a task that has not been executed just now (e.g., CBA task sequence, Arbuthnott and Frank, 2000; Mayr and Keele, 2000). In short, BI means that when a task is switched (A→B), we will inhibit the preceding task A, and this inhibition will still exist after we perform the new task and continue until the subsequent trial. When the next trial is task A that was previously inhibited (A→B→A), the previous inhibition needs to be overcome, leading to behavioral costs, i.e., the cost of deinhibition (Arbuthnott and Frank, 2000; Mayr and Keele, 2000; Grange and Houghton, 2010; Costa and Friedrich, 2012). It can be shown both in the reaction time slowdown and by increase in the number of errors. Therefore, the BI effect was first used to prove that there is a process of inhibiting the previous task in task switching (Mayr and Keele, 2000; Mayr, 2007).

In the past 20 years, more and more researchers have conducted research on the BI effect, but the BI effect specifically reflects which cognitive process is ambiguous. Although researchers have adopted the term BI in the past decades, in our opinion, it is inappropriate to call BI when we check the relevant operational definition and measurement methods. For example, some studies have determined that cognitive deficits (i.e., Parkinson’s disease, Fales et al., 2006), underdeveloped brains (i.e., adolescents, Giller et al., 2019), and aging brain functions (i.e., elderly, Giller and Beste, 2019) will lead to greater BI effects. However, some previous researches believe that the BI effect reflects the intensity of inhibition. The larger the BI effect, the greater the intensity of the inhibition of the previous stimulus (Mayr, 2001; Whitmer and Banich, 2012; Wolff et al., 2018). This seems to be unexplainable. Is it possible that individuals with impaired brain function or underdeveloped brain function have stronger inhibitory capacity? Therefore, it is urgent to clarify the nature of the BI effect and its internal mechanism. In fact, some researchers have pointed out that in BI research, most of the investigations are deinhibition, not the inhibition of previous stimuli (Mayr and Keele, 2000; Dreher and Berman, 2002; Fales et al., 2006; Giller and Beste, 2019; Picazio et al., 2020). Therefore, the first aim of this review is to summarize the main paradigm of BI research, the development of BI ability, and analyze whether the cognitive process reflects the inhibition process or the deinhibition process. In addition, more and more cognitive neuroscientists have found that multiple brain regions, including the frontal lobe, parietal, basal ganglia, and cerebellum, are activated during deinhibition. The ERP studies have shown that deinhibition process is reflected in the P1/N1 and P3 components, which might be related to early attention control, context updating, and response selection, respectively. Although these studies have made great contributions to revealing the brain mechanism of BI, there are still inconsistent conclusions and many problems to be solved. The second aim of this review is to conduct a comprehensive summary and analysis of the research on the neural mechanisms of BI in order to understand the brain mechanisms of BI more clearly. Finally, this article will give a clear conceptual definition of BI to correct the incorrect description of the nature of BI in existing studies.

## Experimental Paradigms

Usually, the three consecutive tasks involved in the triple task paradigm are treated as a single unit. The position where the first task appears is defined as trial N-2, the position where the second task appears is defined as trial N-1, and finally, the current task is regarded as trial N. According to a different sequence, task sequences, such as ABA and CBA, are formed. The BI effect is mainly reflected based on the difference in the response time between ABA and CBA. Based on the dimensions of switching and task requirements, the research paradigms of BI can be divided as follows.

### Perceptual Dimension Switching Task

In the perceptual dimension switching task, participants need to switch between different stimulus dimensions among multiple stimuli (Mayr and Keele, 2000; Grange and Houghton, 2009, 2010; Grange and Juvina, 2015; Kowalczyk and Grange, 2017). In the study of Mayr and Keele (2000), participants were asked to search for the pop-out items among the given four items. The searching criteria included identifying three different dimensions (color, direction, and movement status). Participants were asked to search for items that deviated from the pre-specified dimensions and reacted through the corresponding buttons. In each trial, each item deviated from the whole with respect to only one dimension. For example, if the specified dimension in trial N-2 was color, then the Lag-2 repetitive (or non-repetitive) trials were defined when the target dimension in trial N was the same (or different) as trial N-2. The reaction time (RT) of the Lag-2 repetitive sequence was reported to be longer than the non-repetitive sequence.

### Movement Direction Switching Tasks

In the movement direction switching tasks, participants must move the target directionally according to cues that are repeated or switched across trials (Mayr, 2002; Grange, 2018; Kowalczyk and Grange, 2020). Kowalczyk and Grange (2020) asked the participants to perform a rapid spatial transformation of the stimulus location according to the cues and move the stimulus to the correct location by pressing the key corresponding to the spatial location. The numbers “1,” “2,” “4,” and “5” corresponded to four locations: bottom left, bottom right, top left, and top right, respectively. Three cues corresponded to three types of movement tasks (hexagon: vertical movement; square: diagonal movement; triangle: horizontal movement). For example, if the stimulus appeared in the bottom left, and the task required horizontal movement, the participant needed to move the stimulus to the bottom right by pressing the button “2.” According to three different movement rules, two task sequences, CBA and ABA, were obtained. Kowalczyk and Grange (2020) found that when task A in trial N-2 appeared again (ABA), the response time of task A was significantly slower than task A in the CBA sequence.

### Semantic Dimension Switching Task

Numerous studies on BI have used the semantic dimension switching task, in which participants classified the stimuli based on semantic rules (Dreher and Berman, 2002; Schuch and Koch, 2003; Arbuthnott, 2008; Greenberg et al., 2013; Schuch and Grange, 2015; Jost et al., 2017; Regev and Meiran, 2017; Sexton and Cooper, 2017; Picazio et al., 2020). For example, Schuch and Koch (2003) used numbers 1–9 as stimuli and asked the participants to perform three tasks, i.e., parity (odd number vs. even number), magnitude (small number vs. large number), and distance (near vs. far from 5) judgments. Dreher and Berman (2002) used alphabets to allow the participants to make a judgment regarding vowels/consonants, uppercase/lowercase, and before/after the letter “m” according to different color cues.

### Modality Switching Task

Several recent studies have adopted the modality switching task (Zhang et al., 2016a,^2017^; Wolff et al., 2018; Giller and Beste, 2019; Giller et al., 2019, 2020). In the experiment by Koch et al. (2004), two tasks included numerical judgment tasks (parity and size), and the third task was a simple reaction task, requiring the simultaneous pressing of two reaction keys when the stimulus appears. The square represented task A (odd/even), the diamond represented task B (large/small), and the triangle represented task C (double press). Compared with more complex tasks that required numerical judgment, N-2 repetitive costs also existed in simple reaction tasks. Philipp and Koch (2005) explored the BI effect on different response modes using three cues: square, triangle, and rhombus, which represented pedal response, finger response, and voice response, respectively. Participants were asked to make a judgment on the size or parity of numbers. In the whole experiment, each participant was only asked to make a type of number judgment (size or parity). Participants needed to use the appropriate response mode (manual, sound, or pedal response) based on these cues. It turns out that when switching between different modes, N-2 repetitive costs also existed, which indicated that the BI effect could be observed on task sets with different reaction modes.

## Development of BI and Impairment in Patients

### Adolescents Versus Adults

Compared with the adults, the cognitive flexibility of adolescents is known to be not fully developed (Lehto et al., 2003; Davidson et al., 2006; Hauser et al., 2015), and it is more difficult for adolescents to overcome the BI effect than adults (Giller et al., 2019). Giller et al. (2019) compared the ability of deinhibition (i.e., overcoming BI effect) between the adolescent and adult group. They found that there was no significant difference between the two groups under base conditions (CBA). However, under BI conditions (ABA), the response time of adolescents was significantly longer than adults, leading to a significantly greater BI effect (ABA minus CBA) for adolescents than adults, which suggested that the adolescent group had a lower ability of deinhibition than the adult group.

The underlying neural principle of the development of BI during adolescents was hypothesized to be as follows. The response selection mechanism in the process of deinhibition has been shown to be regulated by the dopaminergic system and related to the activity of the anterior cingulate cortex (Zhang et al., 2016a), and the BI effect is also related to the functions of the basal ganglia, auxiliary motor areas, and premotor areas (Whitmer and Banich, 2012). Adolescents exhibit difficulties in overcoming BI, which could be attributed to the fact that their dopaminergic system and medial frontal brain structure are still underdeveloped (Segalowitz et al., 2011; Simon and Moghaddam, 2017).

### Adults Versus Elderly

The ability to overcome BI effects might also be affected by aging (Mayr, 2001; Giller and Beste, 2019). Mayr (2001) found that the BI effect of the elderly was greater than young adults. BI is known to reflect the intensity of inhibition. Thus, the inhibition intensity of the elderly in the BI tasks would be greater than that of young adults. Giller and Beste (2019) compared the ability of deinhibition between the elderly and young adults. The BI effect of the elderly group was found to be significantly greater than the young adults, indicating that the elderly participants found it more difficult to perform the recently inhibited tasks. The aging of BI has been suggested to be related to the function of the right inferior frontal gyrus (rIFG), which is known to be involved in the inhibitory process (Wolff et al., 2018). Due to the aging or dysfunction of rIFG in the elderly (Cooray et al., 2014; Kleerekooper et al., 2016; Hu et al., 2018), elderly adults are known to be more likely to show a decrease in deinhibition ability as compared to the young adults. The aging of BI might also be related to the dopaminergic system, which would decline during the aging process (Grady, 2008; Eriksen et al., 2009; Uchida et al., 2009).

However, other studies have not found evidence of the aging of BI (Li and Dupuis, 2008; Lawo et al., 2012; Schuch, 2016). For example, Li and Dupuis (2008) investigated the age difference of the BI in the sequential flanker task and found that there was no difference in the size of BI between different age groups. In the study of Schuch (2016), participants were asked to respond to facial expressions, which were evaluated based on gender, mood, and age. Schuch (2016) also showed no significant difference in the BI effect between the elderly and the young adults.

### Impairment of BI in Patients

Fales et al. (2006) allowed the patients with mild to moderate Parkinson’s disease (disease group) and the control group to perform three tasks in a mixed manner. Compared with the control group, the patients in the disease group made a significantly greater number of mistakes during alternate switching (ABA). The author suggested that these PD patients who had difficulties in overcoming BI could not direct their attention to the new task set, i.e., PD patients had disproportionate difficulties in organizing resources to reactivate recently suppressed task settings. Previous studies have also shown that patients with Parkinson’s disease experience difficulty in shifting attention to new tasks (Brown and Marsden, 1988; Meiran et al., 2004; Gul et al., 2017).

Wolff et al. (2018) compared adolescent patients with obsessive-compulsive disorder (OCD) and normal adolescent participants. A major aspect of OCD involves cognitive inflexibility (Chamberlain et al., 2006; Rosa-Alcázar et al., 2020; Martínez-Esparza et al., 2021). Flexible cognitive abilities are sometimes required to re-use thought patterns that have been abandoned recently. Therefore, in some cases, cognitive flexibility can help restore certain stereotyped, repetitive behaviors of OCD. Wolff et al. (2018) demonstrated that compared with the normal group, patients with OCD showed a smaller BI effect. However, the results also showed that the smaller BI effect in patients with OCD was due to the significantly increased RT in the base condition (CBA) rather than the decrease of RTs in the ABA response time. Therefore, the smaller BI effect shown by patients with OCD did not correspond to a better ability of deinhibition.

Based on the above findings, both experimental paradigms and material should be taken into account when exploring the BI effect of special patients. On the one hand, not all task paradigms are adequate to elicit BI. As mentioned above, the inhibition process in the task switching paradigm is implicit, and if the inhibition cannot be successfully activated, the deinhibition ability cannot be clearly observed for the patients with obsessive-compulsive disorder (Wolff et al., 2018). On the other hand, the selection of the material is also important to reveal the BI effect (Foti et al., 2015a,b; Picazio et al., 2020). For example, Foti et al. (2015a; 2015b) demonstrated that Williams syndrome exhibited normal BI effect during verbal task-switching, but no BI effect during visuospatial task-switching, which indicates that the BI effect may be Domain-Specific. In addition to special patients, the reproducibility of BI effect in healthy participants might be also depended on the experimental paradigms and material. BI effect will not occur when the current task only requires bottom-up processing, or the inhibition induced by the previous trial is not sufficient to persist until the current trial (Mayr and Keele, 2000, Experiment 3; Picazio et al., 2020). For example, Picazio et al. (2020) observed no BI effect in the spatial task-switching paradigm. They explained that the visual features of the target stimulus were sufficient to trigger the response in a bottom-up manner with no need of top-down control. Another possible explanation is that spatial task-switching is easier than verbal task-switching. In the case of long RSI (1,500 ms) and stimulus presentation time (2,500 ms), relatively easy task requirements may cause the inhibition induced by the previous trial to fade naturally before entering the current trial, so there is no spatial BI effect. In summary, various task parameters should be considered to ensure the repeatability of BI effects.

## Brain Areas Associated With BI

### Frontal Lobe

Previous neuroimaging studies have shown that the right PFC is activated while resolving the response conflict or response inhibition (Garavan et al., 1999; Konishi et al., 1999; Hazeltine et al., 2000). Mayr et al. (2006) found that the BI effect disappeared in patients with right frontal lobe lesions. Additionally, researchers believe that the BI effect reflects the inhibition of irrelevant tasks. The inhibition function is known to be impaired in patients with right frontal lobe lesions, resulting in the disappearance of the BI effect. The findings of Mayr et al. (2006) were consistent with those of Aron et al. (2004), who reported greater task-set interference in right prefrontal patients after a task switch. These studies indirectly proved that the BI effect was related to the inhibitory function of the right prefrontal lobe. By analyzing the Target-P1 at electrodes PO1/PO2, Giller et al. (2019) found that the difference between BI and basic conditions of young people was greater than the elderly group. Also, the source location analysis showed that the activation difference of the rIFG was related to this effect (Giller and Beste, 2019; Giller et al., 2019).

A series of subsequent brain imaging studies showed that the deinhibition ability exhibited by the frontal lobe was not necessarily related to inhibition. For example, Dreher and Berman (2002) asked participants to make different judgments (vowels/consonants, uppercase/lowercase, or before/after the letter M) on colorful letters that were continuously presented every 2.5 s. They found that, compared with the ABC task sequence, the response time of the ABA task sequence was significantly increased, and the right prefrontal lobe evoked greater activation. Dreher and Berman (2002) indicated that the observed activation of the right prefrontal lobe did not reflect inhibition itself but a response to the residual of inhibition, especially to overcome the residual inhibition of recently inhibited tasks, i.e., deinhibition. In addition, Dreher and Berman (2002) also found that there was no interaction between this effect and motor initiation; thus, overcoming the residual inhibition of recently completed tasks was not dependent on the previous motor response, which proved that the lateral PFC mainly played a role in overcoming the inhibition of the cognitive level rather than the inhibition of the motor level (Dreher and Berman, 2002).

In addition, activation of the pre-supplementary motor area (pre-SMA) located in the frontal lobe was also found to be related to the magnitude of the BI effect. Picazio et al. (2020) used the continuous theta burst stimulation (cTBS) to stimulate the right pre-SMA of the healthy participants and observed a disappearance of the BI effect in the verbal BI task. The target stimulus in this task were animal words, and the participants were instructed to complete three distinct tasks (A: two or four legs; B: small or large animal; C: with or without tail). Notably, the stimulation on pre-SMA did not make speedy the response of the ABA sequence but significantly slowed the response of the CBA sequence. Therefore, further studies are required to verify the role of pre-SMA in deinhibition.

### Parietal Lobe

Although the parietal cortex is known to play an important role in inhibitory control and cognitive flexibility (Hedden and Gabrieli, 2010; Fedorenko et al., 2013; Tang et al., 2016; Tschentscher et al., 2017; Hartung et al., 2021), the role of the parietal cortex in BI is unclear. Sdoia et al. (2020) used transcranial direct current stimulation (tDCS) to stimulate the prefrontal or parietal area and assessed the potential ability of deinhibition reflected as the N-2 task repetition cost. In the frontal stimulation condition, the anode was placed over the right dorsolateral prefrontal cortex (F4 according to 10–20 EEG International System), whereas the cathode electrode was placed over the left dorsolateral prefrontal cortex (F3). In the parietal stimulation condition, the anode was placed over the right parietal site corresponding to P4 and the cathode electrode over P3. Participants were instructed to perform three numerical judgment tasks (A: odd number vs. even number; B: small number vs. large number; C: near vs. far from 5). The results showed that the stimulation of the prefrontal and parietal cortex resulted in a faster response time to the ABA sequence, indicating the improved ability of deinhibition. However, there was no change in the BI effect when only the prefrontal lobe was stimulated. Sdoia et al. (2020) observed improved performance of the CBA sequence, indicating that the stimulation of the prefrontal lobe not only improved the ability of deinhibition but also improved the ability to switch tasks. When only the parietal cortex was stimulated, the BI effect disappeared due to the fact that the performance of the CBA sequence remained unchanged. Therefore, when stimulating the parietal cortex, it did not change the performance of task switching but specifically improved the ability of deinhibition. This study demonstrated that the parietal cortex, other than the frontal cortex, played a specific role in deinhibition.

The findings of Sdoia et al. (2020) were obviously different from that of Dreher and Berman (2002). The discrepancy of the conclusion could be due to the methodological differences, such as stimuli and experimental procedure between these two studies (Sdoia et al., 2020).

### Other Brain Areas

Given the close functional connection between the frontal lobe and the cerebellum (Picazio and Koch, 2015; Bostan and Strick, 2018). Picazio et al. (2020) found that when TBS was applied to the right or left cerebellum, the reaction time of the ABA sequence was accelerated, indicating that the cerebellum had a specific contribution to the process of detecting the position of a single event in the task sequence since the BI effect was observed in the triple task and involved the recognition of the sequence of tasks. The cerebellum played a role by recognizing the repetitive elements in the sequence and making the necessary efforts to prepare the deinhibition of the N-2 task. Interestingly, the cerebellar network is known to be closely related to cognitive flexibility. Studies have shown that it is difficult for patients with cerebellar damage to give up previous correct representations that interfere with the current task (Thach, 1996; De Bartolo et al., 2009; Steffener et al., 2016). Therefore, in the BI task, the cerebellum recognized the necessary sequence, which helped avoid past interference.

The deinhibition also activated the occipital cortex and the left subtemporal area (Cohen et al., 2000). The left subtemporal activation is consistent with the visual area activated by the letter string (Cohen et al., 2000). The activation of these brain regions could reflect the deinhibition at the perceptual level. In other words, if a perceptual feature (e.g., color) related to the task in trial N-2 was inhibited after the task in trial N-1 was completed, the inhibition of this feature continued in the subsequent trial N, increasing the difficulty of performing the same task again.

Moreover, some studies have demonstrated the role of the basal ganglia (BG) in deinhibition (Fales et al., 2006; Markett et al., 2011; Whitmer and Banich, 2012). Fales et al. (2006) found that patients with Parkinson’s disease, which was predominantly BG dysfunction, had abnormal BI effects. Markett et al. (2011) found that the polymorphism of the DRD2 gene could be used to predict an individual’s BI effect, and the DRD2 gene affected the density of D2 receptors in BG (Stelzel et al., 2010). However, Whitmer and Banich (2012) found that participants with high BI scores had greater activation in the basal ganglia (BG) and pre-SMA during task switching than participants with low BI scores. This showed that these brain regions were related to the ability to inhibit the previous task. Parkinson’s disease is also known to affect the function of brain regions other than BG (Fales et al., 2006). Participants with a higher BI score had a stronger BG activation during task switching, which indicated that the inhibition during task switching was connected to deinhibition after switching, and this connection was mainly reflected in BG. However, the specific role of BG in BI condition or deinhibition is still unclear.

## Electrophysiological Correlates of BI

Several studies on task switching have firmly established the role of both proactive and reactive control in task switching (Braver et al., 2007; Kiesel et al., 2010; Braver, 2012). Proactive control mainly includes the related process of task preparation. In behavior researches, it can be manipulated by changing the amount of information and preparation interval (the interval between cue and stimulus) of cues. Reactive control aimed to optimize target processing by minimizing the impact of carryover interference (e.g., backward interference, positive or negative priming) and post-target interference (e.g., irrelevant character priming). Usually, in the task-switching studies, the event-related potentials (ERP) could distinguish the neural activity of the cue-locked and the target-locked process so that it can directly measure the proactive and reactive control in task switching (Grange and Houghton, 2014) and then answer the question regarding whether BI required a proactive control or a reactive control.

### BI Effect in Cue Processing

In the cued task-switching paradigm, participants could prepare for the upcoming task through the presented cues; thus, the proactive control process was reflected by the cue-locked ERP component. The most consistent finding was a relatively positive deflection for task switch compared with task repeat trials, and the switching positivity was maximal over the central and parietal scalp and peaked around 400–600 ms post-cue (Karayanidis et al., 2003; Kieffaber and Hetrick, 2005; Nicholson et al., 2005; Swainson et al., 2006). The switching positivity represents some type of “context updating” or updating of mental representation of the current environment (Donchin, 1981; Verleger et al., 2005; Polich, 2007). Sinai et al. (2007) initially investigated the ERPs correlates of the BI effect. They asked participants to make semantic task judgments on words based on different cues: living or non-living, large or small (relative to a human toddler), or situational task judgments: font color (i.e., words were presented in a red or yellow font for the color task) and screen location (i.e., words were presented at the top or bottom of the screen for the position task). They found that when re-executing the recently inhibited episodic task (high interference), the electrodes on the frontal-central region of the scalp induced a larger positive wave that was similar to P3b under low-interference (i.e., episodic interference) BI conditions, and no cue-induced BI effect was found under high interference conditions. This study showed that the adjustment of the BI effect by proactive control in cue processing was affected by the intensity of interference. When the interference was small (i.e., the need for deinhibition is small), the BI effect was observed in the cue-P3.

When the simpler stimuli and shortened cue-stimulus interval (CSI) were used, the BI effect in the cue processing stage appeared in an earlier time window (Giller and Beste, 2019; Giller et al., 2019, 2020) than that of Sinai et al. (2007). Giller and Beste (2019) found that for young adults, the cue-P1 amplitude was smaller in BI conditions than in the base condition over the parietal sites, whereas for the elderly group, who had a larger BI effect in behavior performance, such cue-P1 effect was not observed. They suggested that the amplitude of cue-P1 reflected the filtration of the relevant stimulus features in the task-related network and the process of inhibiting task-irrelevant information (Klimesch, 2011; Wolff et al., 2017). Thus, in the early classification of incoming sensory information, it was necessary to inhibit task-irrelevant information through inhibition control (Wolff et al., 2018; Giller et al., 2019). The elderly adults had insufficient inhibition control and failed to show the BI effect in cue-P1, which could be one of the reasons for the larger BI effect in behavior.

In addition, Zhang et al. (2016a) found that the presentation of reward reduced the latency of cue-P1 and reduced the BI effect, indicating that the attention process reflected in N1 started earlier in the reward group than the control group. Rewards played a role in the process of cue processing, which promoted the preparation of a task, thereby reducing the BI effect. Zhang et al. (2016b) also found that participants who showed a greater BI effect (poor deinhibition ability) had greater cue-N1 amplitude at the parietal electrode than those with a smaller BI effect. Giller et al. (2019) found similar results in the adolescent group. Adolescents showed a greater BI effect in behavior performance and a greater cue-N1 amplitude than adults, which indicated that for participants with greater BI effects, it was more laborious to refocus on tasks that had been inhibited.

Thus, the appearance of the BI effect in the cue-locked ERP was determined by the stimulus material and the length of the CSI. When the stimulus was simpler, and the CSI was shorter, the BI effect appeared in an earlier time window, with a smaller P1 component in BI condition than base conditions; and for participants with a larger BI effect, it led to a larger N1 amplitude, indicating that it was more laborious to refocus on the recently inhibited tasks. In contrast, when the stimulus was more complex, and the CSI was longer, the BI effect appeared in the later time window (i.e., P3), and the BI condition induced greater P3 amplitudes than the base condition.

### BI Effect in Target Processing

In the study of task switching, the target-locked ERPs evoked by the switch trials were characterized by a larger central N2 component over the frontal sites and a decreased P3 over the central-to-parietal sites. Even under the conditions of sufficient preparation and practice (Whitson et al., 2012), the switch costs still appeared (Karayanidis et al., 2011), which mainly manifested as the larger N2 and smaller P3 in the switch trial (Poulsen et al., 2005; Kopp et al., 2020). Studies on BI found that when the CSI was extended, the BI effect decreased, but when it was reduced to a certain level, it did not change. Therefore, even if the cues were fully processed and the task was adequately prepared, the cost of deinhibition could not be completely eliminated (Mayr and Keele, 2000; Astle et al., 2012), indicating that in addition to proactive control, reactive control also played an important role in the process of overcoming the BI effect.

Until now, only one lab has investigated the target-locked ERPs under short CSI conditions, and no researchers have investigated these ERPs under long CSI conditions. When the CSI was short, the processing of the target also included the proactive control of cue processing. Giller et al. (2019, 2020) found that when the cue was not fully processed, the BI effect was first reflected in the early components, such as the positive waves over the parietal sites and negative waves over the frontal-to-central sites during 100–200 ms after the target presentation (Zhang et al., 2016b,^2017^; Giller and Beste, 2019; Giller et al., 2019). Under the ABA sequence, in trial N-1, the inhibition of the executed task of trial N-2 was regarded as a blocking effect (Giller and Beste, 2019). The inhibition or overcoming of the blocking effect could reduce the interference of BI and thus could successfully overcome the BI effect and could better respond to the recently inhibited task. Therefore, when reacting to the target, stronger inhibition was required under BI conditions to inhibit the blocking effect produced in trial N-1, which was associated with a larger parietal P1 that was considered to reflect the process of inhibiting irrelevant information in the early stimulus classification process (Klimesch, 2011). Source location analysis found that the activity of the right lower frontal gyrus (rIFG/BA47) was related to the above-mentioned P1-blocking effect (Dippel and Beste, 2015; Stock et al., 2016; Bodmer and Beste, 2017; Giller and Beste, 2019). The developmental studies on the BI effect found that, for adults and adolescents, over the posterior parietal sites, the BI condition evoked a larger target-P1 than the base condition, whereas there was no such BI effect in the P1 amplitude for elderly adults (Giller and Beste, 2019; Giller et al., 2020). This finding shows that the elderly could not inhibit the blocking effect by strengthening the inhibitory gating mechanism.

Following the P1 component, a larger N1 was elicited under the BI condition than the base condition (Zhang et al., 2016a,b, 2017; Giller et al., 2019). N1 in the parietal cortex of the brain was thought to reflect attention control, such as concentration on task-related stimuli (Eimer, 1994; Hillyard et al., 1994; Lange, 2012; Marzecová et al., 2017). Participants who showed a larger BI effect in behavioral performance also showed the difference in target-N1 amplitude between BI and base conditions over the parietal sites (Zhang et al., 2016b). These participants had an increased N1 under BI conditions than under base conditions, whereas the smaller BI effect group had no such BI effect in target-N1. Source location analysis found that the BI effect in target-N1 amplitude was associated with the activity of the right parietal lobe, including the temporal-parietal junction, the left cuneiform lingual gyrus, and the parahippocampal gyrus. These brain areas have been shown to be involved in attention modulation and selective attention (Luck et al., 1997; Reynolds and Desimone, 1999; Munneke et al., 2011). Therefore, participants with larger BI effects in behavior performance had reduced ability to re-allocate attention resources to inhibited tasks than the group with a smaller BI effect. It is necessary to note that the group difference in the BI effect was mainly caused by the difference in the base conditions, while the difference in the BI conditions seemed to be non-significant (Zhang et al., 2016b). Thus further studies are required to verify this finding.

BI effect is also reflected in the reaction selection process that is associated with N2/P3 components. Wolff et al. (2018) found that about 500 ms after target presentation, the parietal P3 amplitude in BI condition is smaller than that of base condition and is preceded by a larger N2 (about 350 ms) in BI condition. This ERP result is very similar to the switch-negativity that is associated with reactive control in task switching (Gajewski et al., 2010; Kopp et al., 2020). The switch-negativity in N2/P3 components has been interpreted by the increased difficulty in the decision-making process that is due to the task-irrelevant interference or conflict (Poulsen et al., 2005; Kopp et al., 2020). However, in other ERP studies on BI effects, the seemingly opposite result has been obtained (Zhang et al., 2017; Giller and Beste, 2019; Giller et al., 2020). In addition, Giller et al. (2020) found that the R-cluster, which is considered to reflect the execution process of response, exhibits a higher amplitude in base conditions than BI conditions. The higher amplitude in the base condition may reflect stronger action promotion because there was no inhibition needs to be overcome under the base condition (CBA).

In brief, the extant studies showed that when there is sufficient preparation time to process the cues, there will be a larger switch-positivity in the cue-P3 under BI condition than in the base condition. When the preparation time is short, the difference between BI conditions and base conditions is reflected in the early ERP components (such as P1/N1) post-target presentation, reflecting the early re-allocation of attention and the gating mechanism of inhibition (Zhang et al., 2016a; Giller and Beste, 2019). Some ERP studies support the existence of an obligatory reconfiguration process under the condition of a short CSI; switch-positivity will appear 150–200 ms after the target presentation (Karayanidis et al., 2003; Nicholson et al., 2005). Therefore, in the above-mentioned studies with a short CSI, the BI effect in target-P1 might be due to the forced reconfiguration.

## Conceptual Definition and Theoretical Accounts

**FIGURE 1:**
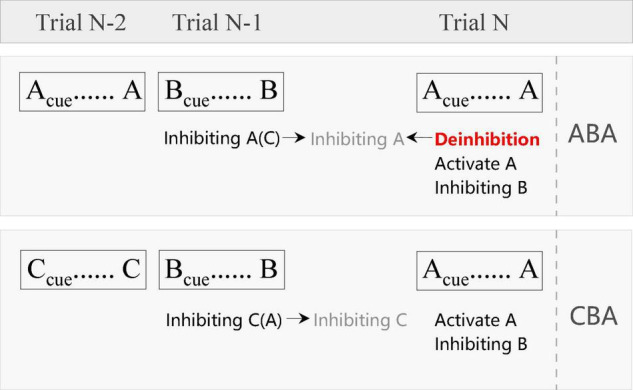
The illustration of the sub-process in the ABA (BI) and CBA (base) condition. Inhibiting A(C) means that both the task A and C should be inhibited when performing task B, while task A needs more inhibition than task C. Inhibiting A (in gray color) means the residual inhibition of task A.

Although researchers have adopted the term “BI” in the past decades, in our opinion, it is inappropriate to call BI when we check the relevant operational definition and measurement methods. As shown in Figure 1, according to the task switching reconfiguration theory proposed initially by Rogers and Monsell (1995), trial N-1 (task B) in ABA can produce an inhibition of trial N-2 (task A), which can last until the current trial N (task A). However, compared with the task B in trial N-1, task A in trial N is a switching trial. Thus, it will be necessary to inhibit the previous task setting and activate the current task setting (Monsell, 2003), that is, to activate task A and inhibit task B. Therefore, in the current trial of ABA sequence, there will be a deinhibition process (i.e., to overcome the residual inhibition of task A), a reactivation of task A, and the inhibition of task B. The potential interrelations between these cognitive processes remain unclear and will be addressed in the future studies. In fact, some researchers have pointed out that in BI research, most of the investigations are deinhibition, not the inhibition of previous stimuli (Mayr and Keele, 2000; Dreher and Berman, 2002; Fales et al., 2006; Giller and Beste, 2019; Picazio et al., 2020). Therefore, the so-called BI reflects the process of removing residual inhibition and reflects the ability of deinhibition. Since most researchers are accustomed to using BI, we also use it in this article, but it should be noted that most of the researchers actually have investigated the process of deinhibition. The concept of deinhibition used in this article comes from biophysical literature (Toselli et al., 1999; Wray et al., 2011; Miao et al., 2019) and the psychiatry studies (Paholpak et al., 2016; Allen and Hallquist, 2020). Taken together, deinhibition refers to the process of removal of or overcoming residual inhibition that is usually named BI effect. Since deinhibition is closely linked to BI, so in the following paragraphs, we would not distinguish them if not necessary.

There are three main theoretical explanations of BI: task setting inertia theory, connection theory, and representational conflict theory. The theory of task setting inertia originated from the discovery of asymmetrical switch costs (Allport et al., 1994). Asymmetrical switch costs refer to the fact that when the participant switches between two tasks of different difficulty, the switch cost of a simple task is greater than that of a difficult task. Asymmetrical switch costs mean that there is proactive interference: task settings will still exist for a period of time after execution, which will affect the switch cost. Accordingly, Allport et al. (1994) proposed the task setting inertia theory, emphasizing the inhibition process during task switching. The reliable evidence for this theory comes from the N-2 repetition cost (Mayr and Keele, 2000; Koch et al., 2010). According to the theory of inertia, the preceding tasks need to be inhibited when switching tasks, especially the tasks that have just been executed need stronger inhibition. This inhibition will continue until a period of time after the completion of the new task. During this duration, if the previously inhibited task should be performed again, participants need to overcome the residual inhibition, which will incur costs.

In the connectionist model, the information that guides the current action is thought to be encoded by densely connected “neuron clusters” (Dehaene and Changeux, 1989) that can maintain performance without external stimuli. Therefore, the control settings tend to remain active for a long time. Connection theory emphasizes bottom-up influence and emphasizes the role of episodic retrieval and connection learning while ignoring the important role of cognitive control in task switching (Abrahamse et al., 2016; Schmidt and Liefooghe, 2016). Grange et al. (2017) extended the study of Mayr (2002) and proved that most of the N-2 task repetition cost could be explained by the non-inhibition process, which supports the episodic retrieval in the connection theory. This episodic retrieval view believes that the cues, target characteristics, and response choices of the task being performed are bound into a single representation and stored in episodic memory. When this task is prompted again, the most recent traces of this task will be automatically retrieved from the episodic memory (Logan, 1988, 2002; Sinai et al., 2007). If the parameters of the current trial are different from the retrieved episodic memory, mismatch costs will occur. According to the episodic retrieval view, if the memory of “not respond to task A” interferes with the current task requirement of “respond to task A,” then context conflict will occur. Therefore, the mismatch of memory trace between task A of trial N and task A of trial N-2 can explain the cost to a large extent (Grange et al., 2017; Grange, 2018).

Sexton and Cooper (2017) proposed a cognitive computational model of BI, which combined the cognitive model of task switching and the cognitive model of conflict monitoring. It is demonstrated that BI is a direct result of conflicts between multiple task representations. This model maps the responses of all tasks to the same set of response buttons. The strength of bottom-up processing in the model can be reflected in the connection weight. For example, compared with the weaker color-naming task, the more advantageous word reading task has a stronger connection weight in the Stroop task. The model makes a clear behavioral prediction of the differential effect of the BI effect. That is, compared with the easy-hard-easy task sequence, there will be a greater BI effect in the hard-easy-hard task sequence. This model argues that BI automatically reduces the interference between multiple tasks, thereby reducing or eliminating deliberate control that needs attention in certain situations and ultimately promotes task control.

Pashler (1984) used the overlapping task paradigm to prove that there is a “bottleneck effect” in cognitive processing. In this paradigm, the subjects were required to respond separately to two distinct overlapping stimuli (SI and S2), with significantly increased RT to both S1 and S2 compared to perform the same task alone. Bottleneck effect refers to the fact that there are certain stages of processing (constituting a bottleneck) that cannot be performed simultaneously on more than one input, and each task that involves the bottleneck must be performed in a specific sequence. BI may also be a possible manifestation of the bottleneck effect, where the parallel cognitive processes of inhibiting task A and activating task A can make the performance of the ABA worse. Dehaene et al. (2003) proposed a neuronal network model based on the “bottleneck effect” to explain the theory of the competitive mental activity. The model assumes that when sensory stimuli enter the global neuronal workspace, it can effectively mobilize the various excitatory neurons with long-distance axons. The neurons that were temporarily mobilized can inhibit other surrounding workspace neurons, which thus become unavailable for processing the other stimuli (Dehaene and Changeux, 2005). From this model, we can hypothesize that in the BI condition, when mobilizing neurons that can inhibit task A, neurons that perform task A will also be inhibited, thereby resulting in N-2 repetition costs. Zylberberg et al. (2010) conducted a computer simulation based on the “bottleneck effect”, further demonstrating that when faced with the dual-task stimuli, neural networks can exhibit parallel processing at the sensory levels and a functional serial bottleneck at the response selection level, whereas the sensory information is held in a memory buffer (Zylberberg et al., 2011). In ABA condition, the inhibition of task A will be in a memory buffer, and when re-reacting to task A, it will have a significant worse performance due to the functional serial bottleneck.

In addition to the above theoretical viewpoints, there are also debates about whether BI is proactive control or reactive control. Some researchers believe that BI is reactive control driven by conflicts between tasks (Koch et al., 2010). However, in recent years, some studies have raised questions about this view, suggesting that BI may be proactive control. Costa and Friedrich (2012) used univalent stimuli and univalent responses to control the conflict between the stimulus level and the response level and tested whether the generation of inhibition must be based on conflict resolution. In this case, if N-2 repetition costs are not found, it means that the inhibition is caused by the conflict resolution process; if N-2 repetition costs are found, it means that the inhibition is caused by leaving the old task to prepare for the new task. The results show that using univalent stimuli and univalent responses still found significant inhibition, indicating that the conflict between the stimuli level and the response level is not a necessary prerequisite for causing inhibition. Deviating from the old task and preparing for the new task can trigger inhibition; that is, BI occurs at the task setting level, which is a kind of proactive control, not reactive control. Gade and Koch (2014) found that there is an inhibitory preparation effect in both abstract and textual cues; that is, N-2 repetition costs decrease with the increase of preparation time, which shows that the inhibition, in this case, is top-down processing.

Finally, we can look at the generation of the cost of deinhibition from the perspective of brain learning. Under hierarchical models of perception, it is necessary to optimize the relative precision of empirical priors and sensory evidence (Kass and Steffey, 1989; Friston, 2009). Neurobiologically, this corresponds to synaptic gain control of the sort invoked for attention. This optimization is crucial for inference. Mechanistically, the role of cholinergic neurotransmission in modulating post-synaptic gain fits comfortably with its role in attention. Therefore, attention might not be the selection of sensory channels but an emergent property of prediction, where high-precision prediction-errors enjoy greater gain (Friston, 2005, 2009). Mehta (2001) pointed out that a critical task of the nervous system is to learn causal relationships between stimuli to anticipate events in the future. Friston et al. (2016) emphasizes a process theory based on active inference and belief propagation, assuming that all neuronal processing (and behavioral choices) can be explained by maximizing Bayesian model evidence. Active inference enables predictions based on the current task state by assuming behavior. Thus, this allows one to define behavior as achieving optimistic predictions dictated by prior preferences (Friston et al., 2014). Expectation states occur frequently as the brain learns to reinforce inhibitory responses to specific stimuli, and actions ensure that prior expectations (stop responding) are met. In the context of task switching, the brain knows in advance which tasks will be faced. When one task is completed, the brain predicts that the probability of another task appearing in the next trial may be greater than that of the task already performed, so that the optimization of task preparation can be achieved by amplifying the expectation of another task by suppressing the executed task. In the case of three tasks, this active inference may be more obvious. For example, the first two trials are tasks A and B, and the brain may expect that the probability of task C will be significantly higher in the next trial. While this expectation is rationally unfounded, the brain does, making it more difficult to respond to task A when it occurs unexpectedly. As found in the findings from the studies on oddball (i.e., unexpected items), both the frontal and occipital lobes of the brain were generally observed to be highly activated when unanticipated and rare stimuli were presented (Brazdil et al., 2007; Hooi et al., 2018), which is overlapped with regions of brain activation found during deinhibition (Dreher and Berman, 2002; Picazio et al., 2020; Sdoia et al., 2020).

## Conclusion and Future Directions

This article reviews the research paradigm, development characteristic, neural mechanism, and the related theories of the BI effect. After summarizing the previous research paradigms and theoretical models involved in BI, first of all, we believe that the cognitive component reflected by the BI effect is deinhibition, rather than the intensity of inhibition mentioned in previous studies. Therefore, in the future, researchers using the concept of BI should pay attention to distinguishing these two connected but functionally different cognitive processes. Second, existing studies have found that the ability of deinhibition is the same as cognitive control. The ability of deinhibition also shows a development process from weak to strong and then declines, but there is still controversy about the development conclusion of its neural mechanism. In the study of brain imaging, the conclusions on the specific brain regions involved in the deinhibition process are still relatively vague. The frontal lobe, parietal lobe, basal ganglia, cerebellum, and other brain areas are activated during the deinhibition process, but there is no definite conclusion. A number of ERP studies have clarified that both the proactive control and response control will work together on the deinhibition process. The deinhibition process occurs from the early stage of attention, and it induces a larger P1/N1 component, indicating that it is more difficult to refocus on tasks that have been inhibited recently, and it also affects the P3b amplitude of the response selection process, the gradual decrease of P3b is consistent with the increasing difficulty of the decision-making process. After systematically summarizing the electrophysiological processes involved in deinhibition, it can be concluded that deinhibition is mainly produced in the early stage of attention processing and the process of response selection. Future research in this field can be explored in the following directions.

### Separate BI and Deinhibition

BI and deinhibition are two concepts that are often indistinguishably used. Although the two are closely related, from the point of view of where it happened, BI stems from the lateral inhibition in trial N-1, while deinhibition occurred in the trial N, which is the removal of the residual inhibition from trial N-1. Therefore, from the perspective of sequence position, these two cognitive processes are separable.

**FIGURE 2:**
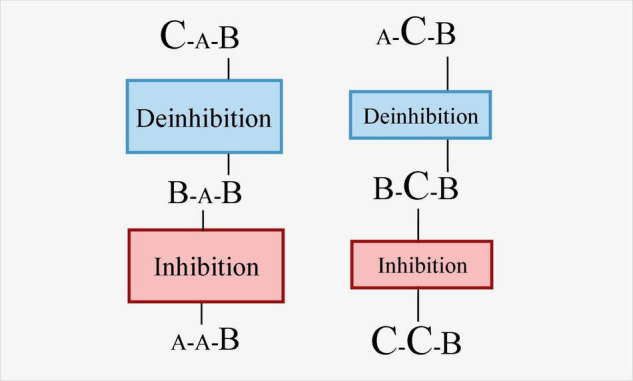
Illustration of the disassociation between inhibition and deinhibition in task sequence with three different tasks of increasing difficulty. The letter with the smallest size indicates that a particular task is easiest, while the letter with largest size indicates that it is the most difficulty one.

In most of the extant studies, the BI and deinhibition processes are studied through three tasks (Mayr and Keele, 2000; Giller et al., 2019). Specifically, the BI effect involves the interaction of trial N-2 and trial N-1 and its influence on trial N (Zhang et al., 2016a,b; Sdoia et al., 2020). When switching back to the most recently inhibited task (ABA) after an intermediate task B, the execution cost is higher than the trial N-1 without inhibition (CBA). This cost is the cost of deinhibition. Therefore, in the three-task paradigm, although BI is focused by many investigators, it is deinhibition other than BI that is measured. Whitmer and Banich (2012) have attempted to separate BI and deinhibition. They divided the participants into high and low groups based on the size of the BI effect, then examined the brain activation during the task switch trials. The results showed that the high BI group had more significant activation in the BG and supplementary motor cortex than the low BI group. Whitmer and Banich (2012) indicated that these areas might be associated with the BI process. This conclusion is simply an indirect inference of BI, not a conclusion drawn from a direct measurement of BI. Future research is suggested to manipulate the inhibitory strength of trial N-1 or manipulate the interference or extent of the intention of overcoming inhibition in the trial N to effectively separate BI and deinhibition. For example, A, B, C are three different tasks with increasing difficulty. According to the studies of switching cost asymmetry (Allport et al., 1994; Gilbert and Shallice, 2002; Yeung and Monsell, 2003), it can be expected that the cost of switching from task B to task A will be greater than the cost of switching from task B to task C. This cost asymmetry has been explained that, a task set remains active even when the task execution is over. Participants must maintain strong inhibition to overcome interference from the easier task when performing the difficult task and such inhibition should be carried over to the succeeding trials, which results in a larger switch cost in switching from the difficult task to the easier task. Accordingly, the inhibition in the process of switching from task B to task A will be greater than that of switching from B to C. As shown in Figure 2, the RT difference between BA and AA might be larger than the RT difference between BC and CC. Because the strength of inhibition was different, the subsequent deinhibition should also be different, that is, the difference in RT between BAB and CAB might be greater than the difference in RT between BCB and ACB. In short, inhibition and deinhibition can be preliminarily separated by comparing the various sequences composed of the various tasks with different difficulty.

### Reveal the Specific Process of Deinhibition

Although studies have shown that both proactive control and reactive control play a role in the deinhibition process, it is still unclear which type of control plays a dominant role. In addition, existing ERP studies have found that the inhibition gating mechanism (P1) and the attention selection mechanism (N1) play an important role in the process of deinhibition. It should be noted that, except for the study of Sinai et al. (2007), the P1 in the other ERP studies mentioned above are all results obtained in a paradigm in which cue and target appear almost simultaneously (Koch et al., 2004). Since the target and the cue are presented almost at the same time (only 100 ms of CSI), it remains unclear whether the target-P1 is completely caused by the targets or is also affected by the preceding cues. Related studies have also found that conflict monitoring plays an important role in the process of deinhibition (Zhang et al., 2017; Giller et al., 2019). But these conclusions are controversial. For example, a study on the deinhibition ability of adolescents and adults found that the C-cluster N2 amplitude of adolescents is smaller under BI conditions than the base condition, but adults do not show this result (Sinai et al., 2007; Giller et al., 2019). In other studies, it has been found that the N2 amplitude of adults under BI conditions will be significantly smaller than the base conditions (Zhang et al., 2016a,^2017^). Therefore, more experiments are needed to reveal the specific sub-processes, such as conflict monitoring in deinhibition.

### Distinguish the Brain Basis of BI and That of Deinhibition

With respect to the brain areas involved in deinhibition, the main finding is that the process of deinhibition locates in the right lateral frontal lobe, cerebellum, and parietal lobe (Dreher and Berman, 2002; Fales et al., 2006; Picazio et al., 2020; Sdoia et al., 2020). However, the materials used in these studies are different, and the results are also inconsistent. For example, Dreher and Berman (2002) used the letter judgment task and found that the right lateral prefrontal lobe played a unique role in deinhibition, while Sdoia et al. (2020) used the number judgment task and found the main brain activation in the parietal cortex. In the study of Sdoia et al. (2020), task conflict occurs at the level of stimulus and response. That is, each task uses the same set of response keys, and the stimulus can trigger all possible tasks. Therefore, it is unclear whether the role of the parietal cortex is related to stimulus processing or response selection in overcoming persistent inhibition. Future research can also explore the different effects of prefrontal and parietal tDCS on the stimulus-related and response-related effects of task inhibition. In addition, whether the cerebellum does play a role in the process of deinhibition, or just to recognize the sequence, also requires further studies.

### Explore the Individual Differences in the Ability of Deinhibition

Research on the development of BI has revealed the group or individual differences in BI and its neural mechanisms. So far, few researchers have investigated the differences in BI among individuals of the same age group. Whitmer and Banich (2012) divided participants into high and low groups based on the size of the BI effect, but they did not report specific behavioral results. Zhang et al. (2016b) also did an individual analysis, but they found that the RT of the base condition is longer for the smaller BI group than for that of the larger BI group, which seemed to indicate that people with stronger deinhibition ability were less flexible when switching tasks. Obviously, this inference may be wrong. Future research needs to confirm whether individuals with different sizes of BI effect processed differently for the task switching trials and further reveal whether these two groups have significant differences in other components of cognitive control such as response inhibition, flexibility, and updating.

It is necessary to note that the ability of deinhibition has been termed one kind of cognitive flexibility, sequential cognitive flexibility (Wolff et al., 2018; Giller and Beste, 2019; Giller et al., 2019), whereas no study has been devoted to specifying the differences between deinhibition and the flexibility in switching from one task to another task. Switch cost is a typical index in measuring the cognitive flexibility in task switching (Monsell, 2003; Philipp and Koch, 2005; Zhuo et al., 2021a,b; Chen, 2022), it can be calculated by subtracting the mean reaction time (or error rate) of the repeat trials from that of the switch trials (Allport et al., 1994; Monsell, 2003; Philipp et al., 2008). Most recently, Chen (2022) conducted a correlation study by comparing the RT cost in a cued switching task and the deinhibition ability in a stop-signal task. They found that these two abilities positively correlated with each other. Future research should directly address this issue in the same paradigm, BI task, to explore whether the individuals who have smaller switch cost in switching from task A to B also have higher ability in deinhibition.

## Author Contributions

JC: primary writing. SW: translation. FL: review and editing. All authors contributed to the article and approved the submitted version.

## Conflict of Interest

The authors declare that the research was conducted in the absence of any commercial or financial relationships that could be construed as a potential conflict of interest.

## Publisher’s Note

All claims expressed in this article are solely those of the authors and do not necessarily represent those of their affiliated organizations, or those of the publisher, the editors and the reviewers. Any product that may be evaluated in this article, or claim that may be made by its manufacturer, is not guaranteed or endorsed by the publisher.

## Abbreviations

BI: backward inhibition
RT: reaction time
PFC: prefrontal cortex
rIFG: right inferior frontal gyrus
Pre-SMA: pre-supplementary motor area
BG: basal ganglia
TBS: theta-burst stimulation
tDCS: transcranial direct current stimulation
PD: Parkinson’s disease
OCD: obsessive-compulsive disorder
ERP: event-related potential
CSI: cue-stimulus interval.
